# Ochronosis as an unusual cause of valvular defect: a case report

**DOI:** 10.1186/1752-1947-3-9302

**Published:** 2009-11-27

**Authors:** Andreas Wilke, Dietmar Steverding

**Affiliations:** 1 Kardiologische Praxis Papenburg, Papenburg, Germany; 2 BioMedical Research Centre, School of Medicine, Health Policy and Practice, University of East Anglia, Norwich, UK

## Abstract

**Introduction:**

Alkaptonuria (also known as ochronosis) is a genetic disorder characterised by the accumulation of homogentisic acid deposits in connective tissue. In rare cases, ochronosis can cause valvular heart disease.

**Case presentation:**

We present the case of a 68-year-old Caucasian man with alkaptonuria-associated degenerative valvular defects with aortic, mitral and tricuspid valve insufficiency. The patient did not have any cardiac complaints and was referred to our clinic for evaluation of a conspicuous new heart murmur.

**Conclusion:**

This case report shows that early diagnosis of cardiovascular ochronosis gives us the opportunity to use conservative treatment to slow down the progression of valvular dysfunction.

## Introduction

Alkaptonuria is a rare autosomal-recessive metabolic disorder characterised by the deficiency of homogentisic 1,2-dioxygenase (HGO) [1]. Due to the lack of this enzyme, homogentisic acid cannot be metabolised and is deposited within various tissues of the body as a polymerised product. The common clinical manifestations of alkaptonuria are (i) homogentisic aciduria, (ii) ochronosis (deposition of bluish-black pigment in all connective tissues), and (iii) arthritis. While alkaptonuria itself is asymptomatic, ochronosis usually develops in the fourth decade of life. There is no cure for alkaptonuric ochronosis and the aim of treatment is to prevent complications.

## Case presentation

A 68-year-old Caucasian man was referred to our cardiology clinic in February 2008 for further evaluation of a conspicuous new heart murmur. The patient did not have any cardiac complaints and did not suffer from angina pectoris or dyspnoea. The patient had advanced gonarthrosis of the left knee, advanced degeneration of the cervical spine and advanced bilateral omarthrosis. His medical history included kidney stone surgery (1988), diagnosis of ochronosis based on a biopsy of the knee joint (1995), total hip replacement (1997), Miller-Galante II prosthesis of the right knee (1997), periosteal rupture of the left Achilles tendon with transosseous re-fixation (1999), ventral corporectomy at C4 and discectomy at C3/C4 and C4/C5 after cervical spinal canal stenosis with myelopathy at C3-C5, and ventral and dorsal osteochondrosis (2001). Family history revealed that his brother also had ochronosis. The patient was not on any medication.

**Figure 1:**
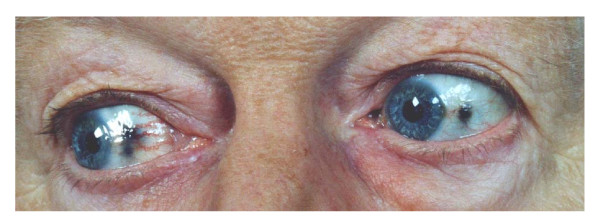
**Ochronotic pigment on the sclera of the eyes of the patient**.

On physical examination, the patient was found to be in a moderately reduced general condition and in a regular nutritional status. His body mass index was 25.8 kg/m^2^. He had a blood pressure of 130/80 mmHg bilaterally and a pulse rate of 80 beats/min. Dyspnoea, cyanosis and liver skin spots were not observed. Bluish-black pigmentations were found on several parts of the sclera (Figure 1). The patient's pupils were of average width and showed prompt response to light. No arcus lipoides, no goitre and no superior vena cava syndrome were noticed. His thorax and chest expansion were symmetrical. Breath sounds were vesicular and percussion resonant, with no crepitations or evidence of a pleural effusion. His heart beat was regular with a grade 2/6 diastolic murmur at the apex and a grade 2/6 systolic murmur over the mitral and tricuspid valves. His abdomen was soft and non-tender to palpation, liver and spleen were not enlarged, and there was no costo-vertebral-angular tenderness. Unilateral oedema on the left ankle was observed. Also, the left foot pulse was absent, while normal central and peripheral pulses were symmetrically palpable.

Results of the patient's laboratory examination showed 252 mg/dl total cholesterol, 84 mg/dl triglycerides, 66 mg/dl high-density lipoprotein and 72 mg/dl low-density lipoprotein. Inflammatory markers were not elevated and anti-streptolysin O (ASO) titre was not raised. The patient's urine turned brownish black when left standing for some time.

An electrocardiogram (ECG) test showed a sinus rhythm of 72 beats/min with left axis deviation (-57 degrees). The patient's depolarisation and repolarisation phases were normal. During an exercise ECG using a treadmill set up to 125 watts workload, his blood pressure increased from 160/90 mmHg to 160/100 mmHg and his pulse rate from 68 to 158 beats/min. When the exercise ECG was stopped at the point of exhaustion, no angina pectoris, dyspnoea, significant ST segment depression or profound dysrhythmia were observed. His blood pressure and pulse rate normalised within 3 minutes of recovery.

**Figure 2:**
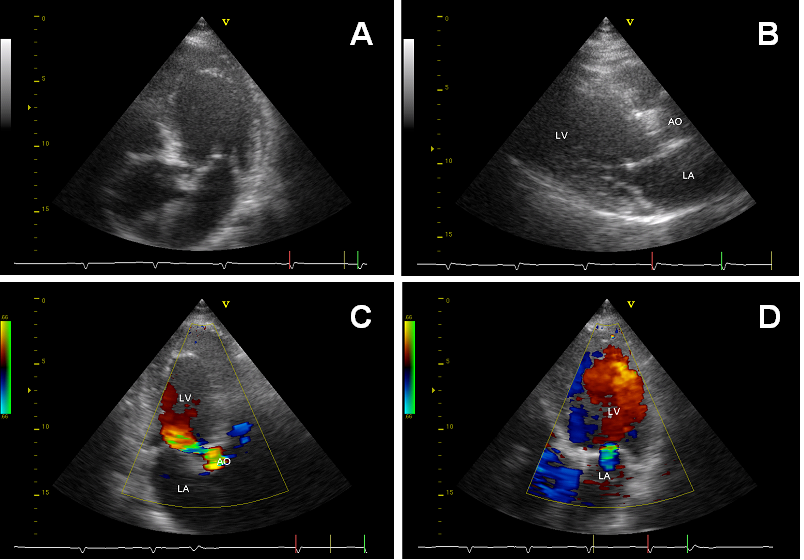
**Echocardiogram of the patient**. **(A)** Apical four-chamber view with aorta. **(B) **Parasternal long axis view with the root of the aorta, left atrium and left ventricular outflow tract. Both images show moderate calcification of the aortic valve and slight calcification at the anterior leaflet of the mitral valve. **(C) **Apical two-chamber view with aorta. Colour flow mode indicates moderate aortic regurgitation. **(D) **Apical four-chamber view. Colour flow mode indicates mild mitral regurgitation.

An echocardiogram revealed that the patient's left atrium was normal in size while his left ventricle was slightly dilated (Figure 2). The pumping function of the left heart was normal - there was no evidence of hypertrophy - and the aortic valve had three cusps. A Doppler echocardiogram showed a minor to moderate aortic insufficiency, a combined mitral valve defect with an opening size of 1.6 cm^2 ^with mild to moderate regurgitation, and a mild tricuspid regurgitation. No pericardial effusion was detected. The patient's right heart was normal in size without any signs of stasis.

A histopathological analysis of the patient revealed brownish deposits between the collagen fibre bundles of a knee joint biopsy sample. The pigment was found both extracellularly and intracellularly. The appearance of the pigment was variable, being coarse, like haemosiderin and fine, like granules. Iron staining was negative and no inflammatory or post-inflammatory changes were observed. Taken together, the patient's histopathology was characteristic for ochronosis.

Based on cardiac investigations, an alkaptonuria-associated degenerative valve defect with aortic, mitral and tricuspid valve insufficiency was diagnosed. The decision for using conservative therapy was reached and the patient was treated with an angiotensin-converting enzyme (ACE) inhibitor (2.5 mg/day ramipril).

At follow-up examinations in April and November 2008, the patient presented in a stable condition. However, in February 2009, the patient was hospitalised because of cardiac decompensation.

## Discussion

Alkaptonuria occurs with a very low prevalence rate of one in 100,000 to 250,000 in most countries with the notable exception in Slovakia, where the disease appears in 1 in 19,000 people [2]. The disease was even detected in an Egyptian mummy [3] and from mediaeval times there are descriptions of the disease which were, however, misinterpreted at that time. In 1866, Rudolph Virchow published the first original description of the disease [4].

Alkaptonuria is an autosomal-recessive inherited genetic disorder of the tyrosine metabolism. Considering conserved synteny, the HGO gene could be mapped to chromosome 3q in six alkaptonuria pedigrees of Slovak origin [5]. So far, 67 different mutations in the HGO gene have been identified [6]. The deficiency of HGO results in the accumulation of homogentisic acid followed by an increased excretion of the metabolite [1]. At first, the urine has a normal colour but after a couple of hours it turns dark due to the high level of homogentisic acid. The ochronotic pigment results from oxidative polymerisation of homogentisic acid [7]. We did not perform a biochemical analysis to determine whether the level of homogentisic acid was elevated but diagnosed the disease based on histopathological and clinical examinations.

In 20% of cases, alkaptonuria is diagnosed before one year of age, while in 80% the mean age at the time of diagnosis is 29 [6]. In general, the severity of the disease progresses after the age of 30 and more rapidly in men than in women [6]. The diagnosis of alkaptonuria is usually based on the detection of degenerative joint disease, ochronosis of the connective tissue and the darkening of urine after alkalinisation. In addition, alkaptonuria may be associated with nephrolithiasis, which develops at a mean age of 64 [6] and could be the leading symptom. Clinical findings include the pigmentation of the ear cartilage and the sclera of the eyes, which only occur after the age of 30 and are very variable in appearance [6,8]. Almost all patients suffer from arthritis of the knee and hip and, occasionally, of the shoulder. By the age of 55 years, 50% of patients with alkaptonuria have undergone at least one joint replacement [6]. However, alkaptonuria-associated arthritis is clinically and radiologically similar to osteoarthritis for which it can be mistaken [9]. Arthritis in patients with alkaptonuria is caused by chemical irritation of deposited homogentisic acid, an altered metabolism of chondrocytes and an altered cross linkage of collagen [10]. Our patient showed most of the histopathological and clinical symptoms, and the final diagnosis was made on the basis of a biopsy taken from his knee joint.

Alkaptonuria is occasionally associated with aortic stenosis but generalised arteriosclerosis and calcification of the heart valves have also been described [11-15]. Cardiovascular ochronosis is usually a benign disease. The disposition of ochronotic pigment serves as a trigger for the dystrophic calcification of valves. Generally, the pigment is deposited at the edge and annulus of valves and may lead to degeneration of cells [11]. However, in our patient's case the histological analysis of valvular material was not possible as there was no indication for valve replacement. Other conditions that could have caused valvular heart disease in our patient are rheumatic fever, endocarditis, age-related calcification of the valves, cardiac dilation and congenital malformations. In patients diagnosed with alkaptonuria, the aortic valve is most frequently affected, followed by the mitral and pulmonary valves. It is also possible that more than one valve is affected. As in our patient's case, aortic and mitral valve regurgitation have been previously observed [12,13]. Alkaptonuria-associated valvular heart disease usually becomes clinically apparent in middle-aged patients (mean age 54 years [6]). Thus, our patient, at 68 years old, was somewhat older for an initial diagnosis of cardiac ochronosis.

Although there is no effective treatment for alkaptonuria [16], the prognosis is relatively good. Large doses of vitamin C (1 g) have been used to prevent the deposition of ochronotic pigment [17]. Nitisinonen is an inhibitor of 4-hydroxyphenylpyruvate dioxygenase, the enzyme that produces homogentisic acid. Nitisinonen has been shown to reduce drastically the production and urinary excretion of homogentisic acid in patients with alkaptonuria [6,18,19]. As the gene responsible for alkaptonuria has been identified, replacement therapy with recombinant enzyme could be a new approach in the treatment of the disease. Alkaptonuria-associated arthritis is usually treated with non-steroidal anti-inflammatory drugs. On the other hand, the prognosis of alkaptonuria is restricted if there are cardiovascular complications. In our case, we only saw indications for treatment with ACE inhibitors, prophylaxis of endocarditis, and annual follow-up. We opted for conservative treatment because of the enlargement of the left ventricle and the assumption that aortic regurgitation would be more severe than mitral regurgitation. In addition, ACE inhibitor treatment is widely recommended in moderate to severe aortic regurgitation [20].

## Conclusion

Bacterial endocarditis, rheumatic fever and valvular sclerosis are the usual causes of valvular heart disease. Our case report shows that a metabolic disorder like alkaptonuria can also be a rare cause of valvular disorder. The bluish-black pigmentation of the sclera (indicative of ochronosis) and the heart murmur (signifying a valvular problem) were the initial diagnostic clues for this case of alkaptonuria-induced valvular heart disease. As cardiovascular ochronosis often requires the replacement of affected heart valves, early diagnosis is important in the morbidity and mortality of the disease. Conservative therapy using ACE inhibitors, as used in conventional aortic regurgitation, may slow down the progression of valvular dysfunction. In summary, although cardiovascular ochronosis is a rare complication in alkaptonuria, patients with this metabolic disorder should be examined for this condition.

## List of abbreviations

ACE: angiotensin-converting enzyme; ASO: anti-streptolysin O; ECG: electrocardiogram; HGO: homogentisic 1,2-dioxygenase.

## Consent

Written informed consent was obtained from the patient for publication of this case report and any accompanying images. A copy of written consent is available for review by the Editor-in-Chief of this journal.

## Competing interests

The authors declare that they have no competing interests.

## Authors' contributions

AW was responsible for patient care and data collection. DS drafted the paper. Both authors read and approved the final manuscript.

## References

[B1] Van Offel JF De Clerck LS Francx LM Stevens WJ The clinical manifestations of ochronosis: a review Acta Clin Belg 1995 50 358 362 8571731 10.1080/17843286.1995.11718475

[B2] Zatková A de Bernabé DB Poláková H Zvarík M Feráková E Bosák V Ferák V Kádasi L de Córdoba SR High frequency of alkaptonuria in Slovakia: evidence for the appearance of multiple mutations in HGO involving different mutational hot spots Am J Hum Genet 2000 67 1333 1339 11017803 10.1016/s0002-9297(07)62964-4 PMC1288576

[B3] Lee SL Stenn FF Characterization of mummy bone ochronotic pigment JAMA 1978 240 136 138 10.1001/jama.240.2.136 351220

[B4] Virchow R Ein Fall von allgemeiner Ochronose der Knorpel und knorpelähnlichen Theile Virchows Arch 1866 37 212 219 10.1007/BF01935634

[B5] Janocha S Wolz W Srsen S Srsnova K Montagutelli X Guénet JL Grimm T Kress W Müller CR The human gene for alkaptonuria (AKU) maps to chromosome 3q Genomics 1994 19 5 8 10.1006/geno.1994.1003 8188241

[B6] Phornphutkul C Introne WJ Perry MB Bernardini I Murphey MD Fitzpatrick DL Anderson PD Huizing M Anikster Y Gerber LH Gahl WA Natural history of alkaptonuria N Engl J Med 2002 347 2111 2121 10.1056/NEJMoa021736 12501223

[B7] Zannoni VG Lomtevas N Goldfinger S Oxidation of homogentisic acid to ochronotic pigment in connective tissue Biochim Biophys Acta 1969 177 94 105 4976426 10.1016/0304-4165(69)90068-3

[B8] Touart DM Sau P Cutaneous deposition diseases. Part II J Am Acad Dermatol 1998 39 527 544 10.1016/S0190-9622(98)70001-5 9777759

[B9] Gupta A Jayanti A Prasanna K Premature arthritis in an elderly woman Int J Clin Pract 2006 60 858 860 10.1111/j.1742-1241.2006.00600.x 16858758

[B10] Gaines JJ Jr The pathology of alkaptonuric ochronosis Hum Pathol 1989 20 40 46 10.1016/0046-8177(89)90200-1 2643557

[B11] Gaines JJ Jr Pai GM Cardiovascular ochronosis Arch Pathol Lab Med 1987 111 991 994 3632274

[B12] Kim YI Daenen W Aortic valve replacement in cardiac ochronosis Eur J Cardiothorac Surg 1992 6 625 626 10.1016/1010-7940(92)90139-O 1449816

[B13] Erek E Casselman FR Vanermen H Cardiac ochronosis: valvular heart disease with dark green discoloration of the leaflets Tex Heart Inst J 2004 31 445 447 15745303 PMC548253

[B14] Hangaishi M Taguchi J Ikari Y Ohno M Kurokawa K Kotsuka Y Furuse A Aortic valve stenosis in alkaptonuria. Images in cardiovascular medicine Circulation 1998 98 1148 1149 9736602 10.1161/01.cir.98.11.1148

[B15] Butany JW Naseemuddin A Moshkowitz Y Nair V Ochronosis and aortic valve stenosis J Card Surg 2006 21 182 184 10.1111/j.1540-8191.2006.00207.x 16492283

[B16] Roser M Möller J Komoda T Knosalla C Stawowy P Alkaptonuric aortic stenosis Eur Heart J 2008 29 444 10.1093/eurheartj/ehm406 17901078

[B17] Mayatepek E Kallas K Anninos A Müller E Effects of ascorbic acid and low-protein diet in alkaptonuria Eur J Pediatr 1998 157 867 868 10.1007/s004310050956 9809834

[B18] Fisher AA Davis MW Alkaptonuria ochronosis with aortic valve and joint replacements and femoral fracture: a case report and literature review Clin Med Res 2004 2 209 215 10.3121/cmr.2.4.209 15931360 PMC1069096

[B19] Suwannarat P O'Brien K Perry MB Sebring N Bernardini I Kaiser-Kupfer MI Rubin BI Tsilou E Gerber LH Gahl WA Use of nitisinone in patients with alkaptonuria Metabolism 2005 54 719 728 10.1016/j.metabol.2004.12.017 15931605

[B20] Mahajerin A Gurm HS Tsai TT Chan PS Nallamothu BK Vasodilator therapy in patients with aortic insufficiency: a systematic review Am Heart J 2007 153 454 461 10.1016/j.ahj.2007.01.006 17383279

